# Supplementary material for: COVID-19 Vaccine Acceptance and Uptake among Healthcare Workers in Trinidad and Tobago
Source: J Environ Public Health. 2022 Sep 9;2022:5031202. doi: 10.1155/2022/5031202 (PMC9481343; doi:10.1155/2022/5031202)
Supplement: Supplementary Materials — Questionnaire. [file 5031202.f1.docx]

* Required

# I have read the foregoing information, or it has been read to me. Do you agree and give consent to voluntarily participate in this research study? *

## Mark only one oval.

Yes No

# Are you currently employed with the NCRHA? *

## Mark only one oval.

Yes No

1. What is your age? *

## Mark only one oval.

18 - 24 years

25 - 34 years

35 - 44 years

45 - 54 years

55 - 64 years

65 - 74 years

75 - 84 years

≥ 85 years

# What is your sex? *

## Mark only one oval.

Male Female

# Regarding your current employment in the NCRHA, please select which title best applies to you from the following: *

## Mark only one oval.

Medical Practitioner Registered Nurse

Enrolled Nursing Assistant

Patient Care Assistant

Paramedic

Pharmacist

Dentist

Veterinary Surgeon Medical Intern Dental Intern

Other:

# What is your monthly income in TTD? *

## Mark only one oval.

< 5,000

5,001 - 10,000

10,001 - 15,000

15,001 - 20,000

20,001 - 30,000

30,001 - 40,000

>40,000

# What is your highest level of education? *

## Mark only one oval.

No formal education Trade school

Primary level Secondary level Bachelor's degree Postgraduate degree

# Did you have any role in the vaccination process at any of the NCRHA vaccination sites? *

## Mark only one oval.

Yes

No *Skip to question 10*

# What was your role at the vaccination site/s? You may select more than one role. *

*Mark only one oval per row.*

Yes No

Patient screening/registration Vaccine Administration Patient Observation

Collating vaccine forms

Yes *Skip to question 12*

No, but I am willing to take a brand of the vaccine *Skip to question 14*

No, I am not willing to take any brand of the vaccine

# Please explain your decision to refuse or to delay receiving a COVID-19 vaccine (select all that apply): *

*Mark only one oval per row.*

Yes No

I am confident there will be other effective treatments soon

I do not yet know enough about the COVID- 19 vaccine to make a decision

I want to gain natural immunity to the virus that causes COVID-19

Development of the COVID-19 vaccine was rushed / vaccine was not thoroughly tested prior to approval

I believe vaccines may give you the disease they are designed to protect against

I don't know

*Skip to question 18*

# How many doses have you received thus far? *

## Mark only one oval.

First dose of a two-dose vaccine

Both doses of a two-dose vaccine

One dose of a one-dose vaccine

# Which COVID-19 vaccine did you receive? *

## Mark only one oval.

Oxford-AstraZeneca Sinopharm

Pfizer-BioNTech Johnson & Johnson Other

If you have taken the COVID-19 vaccine, certain factors must have motivated you to do so. If you are waiting for your turn to get vaccinated, then certain factors might be responsible for your decision to take the vaccine. Below are some statements regarding this. Please indicate the response which you believe best explains your opinion on each of the following statements. If using a mobile phone, please scroll to the right to see the full spectrum of options.

1. I have taken/will take the COVID-19 vaccine because... *

*Mark only one oval per row.*

There is no harm in taking the vaccine.

The vaccine will be useful in protecting me from COVID-19 infection.

The vaccine is available free of cost.

My healthcare provider/doctor recommended it to me.

I am an at-risk individual because of my underlying health problems.

The benefits of taking the vaccine outweigh the risks involved.

The presence of different COVID-19 variants has made me more wary of the COVID-19 virus.

The most recent surge in COVID-19 cases and deaths in the country has caused me to become wary of the virus.

Taking the vaccine is a societal responsibility.

COVID-19 vaccination will help us return to normal activity sooner.

Strongly disagree

Disagree Neither agree

nor diasgree

Agree Strongly agree

There is sufficient data regarding the vaccine's safety and efficacy released by the government and being disseminated locally.

Many people including my peers are taking the COVID- 19 vaccine.

It will help in eradicating COVID-19 infection.

My role models/political leaders/senior doctors/scientists have taken the vaccine.

# Of the COVID-19 vaccines available in Trinidad & Tobago, which vaccine would you prefer/would have preferred to take? *

## Mark only one oval.

Oxford-AstraZeneca Sinopharm

Pfizer-BioNTech Johnson & Johnson

No preference

# If there were no limits on COVID-19 vaccine availability in Trinidad & Tobago, which of the following COVID-19 vaccines would you prefer to take in the future? *

## Mark only one oval.

Pfizer-BioNTech *Skip to question 17* Oxford-AstraZeneca *Skip to question 17* Moderna *Skip to question 17*

Johnson & Johnson *Skip to question 17*

Sinopharm *Skip to question 17* Sinovac *Skip to question 17* Sputnik V *Skip to question 17*

No preference *Skip to question 18*

Other *Skip to question 17*

# Why do you prefer this particular brand of COVID-19 vaccine? Select all that apply. There are no right or wrong answers. *

*Mark only one oval per row.*

Yes No

Vaccine effectiveness

I trust the manufacturer

Transparency and availability of information about this vaccine

Lower incidence of side effects

I know someone/multiple people who took this vaccine and is/are fine now

Sufficient clinical trials were conducted to test this vaccine

It is an approved vaccine for travel to a country I want to visit

1. Please select from the following, the sources of information which have informed your opinion about COVID-19 vaccination (select all that apply). *

*Mark only one oval per row.*

Yes No

National newspapers

International newspapers

National television broadcasts

International television broadcasts

National radio broadcasts

Internet

Social media (Facebook, Instagram, WhatsApp, Twitter, TikTok & YouTube)

Your doctor/other healthcare professional Government agencies

International organizations (WHO, CDC, others)

Family and friends

# On the scale below, how much do you trust COVID-19 vaccine information from the following sources? *

| *Mark only one oval per row.* | No trust | A little trust | Moderate trust | A lot of trust |
| --- | --- | --- | --- | --- |
| National newspapers |  |  |  |  |
| International newspapers |  |  |  |  |
| National television broadcasts |  |  |  |  |
| International television broadcasts |  |  |  |  |
| National radio broadcasts |  |  |  |  |
| Internet |  |  |  |  |
| Social media (Facebook, Instagram, WhatsApp, Twitter, TikTok & YouTube) |  |  |  |  |
| Your doctor/other healthcare professional |  |  |  |  |
| Government Agencies |  |  |  |  |
| International organizations (WHO, CDC, others) |  |  |  |  |
| Family and friends |  |  |  |  |

1. Would you recommend the COVID-19 vaccine to the public? *

## Mark only one oval.

Yes

No *Skip to question 22*

# Which brand of vaccine would you recommend to the general public? *

## Mark only one oval.

Oxford-AstraZeneca Sinopharm

Pfizer-BioNTech Johnson & Johnson

Whichever brand is available

The only brand I am willing to recommend is not currently available to the general public of Trinidad & Tobago

# Would you advise parents to have their children 12 years and older take the Pfizer- BioNTech vaccine? *

## Mark only one oval.

Yes No

# Would you advise your child, or family members who meet the age eligibility in T&T, to take the Pfizer-BioNTech vaccine? *

## Mark only one oval.

Yes No

The questions below include statements about COVID-19 vaccination. Please select the response which best suits your opinion regarding each statement.

1. Healthcare employers must ensure that their employees are COVID-19 vaccinated

*

## Mark only one oval.

Strongly agree

Agree

Neither agree nor disagree Disagree

Strongly disagree

# It is within an employee's rights to refuse COVID-19 vaccination. *

## Mark only one oval.

Strongly agree

Agree

Neither agree nor disagree Disagree

Strongly disagree

# It is within an employee's rights to refuse to supply their employer with their COVID-19 vaccination status. *

## Mark only one oval.

Strongly agree

Agree

Neither agree nor disagree Disagree

Strongly disagree

# It is within an employer's rights to terminate an employee who refuses the COVID- 19 vaccine. *

## Mark only one oval.

Strongly agree

Agree

Neither agree nor disagree Disagree

Strongly Disagree

# Patients have a right to a COVID-19 vaccinated healthcare provider. *

## Mark only one oval.

Strongly agree

Agree

Neither agree nor disagree Disagree

Strongly disagree

# The only COVID-19 vaccine I am willing to take is not currently available in Trinidad & Tobago. *

## Mark only one oval.

Strongly agree

Agree

Neither agree nor disagree Disagree

Strongly disagree

# The Oxford-AstraZeneca vaccine will be effective against future variants. *

## Mark only one oval.

Strongly agree

Agree

Neither agree nor disagree Disagree

Strongly disagree

# The Sinopharm vaccine will be effective against future variants. *

## Mark only one oval.

Strongly agree

Agree

Neither agree nor disagree Disagree

Strongly Disagree

# The Pfizer-BioNTech vaccine will be effective against future variants. *

## Mark only one oval.

Strongly agree

Agree

Neither agree nor disagree Disagree

Strongly Disagree

# The Johnson & Johnson vaccine will be effective against future variants. *

## Mark only one oval.

Strongly agree

Agree

Neither agree nor disagree Disagree

Strongly Disagree

# More brands of COVID-19 vaccines should be made available in Trinidad & Tobago

*

## Mark only one oval.

Strongly agree

Agree

Neither agree nor disagree Disagree

Strongly Disagree

There may be several concerns regarding the COVID-19 vaccine that may influence or may have influenced your decision to take OR not to take the vaccine. Give your opinion on how the following statements have influenced/will influence your decision on whether or not to take the COVID-19 vaccine. If using a mobile phone, please scroll to the right to see the full spectrum of options.

1. I am/was concerned that... *

*Mark only one oval per row.*

The vaccine might not be easily available to me.

There is a risk of contracting the COVID-19 virus from taking the vaccine

The benefits of taking the vaccine do not outweigh the risks of taking it

I might have serious side effects immediately after taking the vaccine

I might have some unforeseen future effects of the vaccine

There is not enough global information regarding the vaccine

There is not enough local information regarding the vaccine

There is not enough global transparency with regards to side effects of the vaccine

There is not enough local transparency with regards to side effects of the vaccine

Strongly disagree

Disagree Neither agree

nor disagree

Agree Strongly Agree

The vaccine has only been granted Emergency Use Approval and not Full Approval by the WHO/FDA

The vaccine may be faulty or fake

The vaccine has not been proven effective

The vaccine was rapidly developed and approved

The vaccine is being promoted for commercial gains of pharmaceutical companies
